# Melatonin ameliorates cisplatin-induced neurodegeneration in medulla oblongata through the expressions of Aqp-1,-4, inflammation, and apoptosis pathway genes

**DOI:** 10.3906/biy-2110-90

**Published:** 2022-01-10

**Authors:** Özlem ÖZTOPUZ

**Affiliations:** Department of Biophysics, Faculty of Medicine, Çanakkale Onsekiz Mart University, Çanakkale, Turkey

**Keywords:** *aquaporin-1*, *aquaporin-4*, cisplatin, medulla oblongata, melatonin, neuroprotection

## Abstract

In this study, the neuroprotective effects of melatonin (MEL) with changes in apoptosis, inflammation, and histopathological morphology were evaluated in the medulla oblongata of cisplatin (CIS) administered rats. Although the side effects of CIS are known in many tissues, its reaction on the medulla oblongata and the molecular association underlying this effect is unclear. Male wistar albino rats were separated into four groups (control, CIS, CIS+MEL, and MEL) (n = 24). CIS and CIS+MEL groups were given 4 mg/kg CIS at 4-day intervals (days 1, 5, 9, and 13) by the first day of the study. The MEL and CIS+MEL groups were given 10 mg/kg MEL daily for 13 days. At the end of the study, the medulla oblongata sections of the rats were harvested on the 14th day, and the changes in gene expressions were examined. Expression levels of inflammation markers (*TNF-α* and *IL-6*), apoptotic markers (*Bax* and *Casp-3*), and *Aqp-1* and *Aqp-4* were found to significantly increase with CIS administration. On microscopic examination, hemorrhage, edema, and perivascular edema were detected in the CIS applied group compared with controls. MEL treatment significantly reduced perivascular edema (p = 0.0152) and hemorrhage (p = 0.0087). Besides, there was a significant difference between the control and CIS groups regarding pyknosis and a significant increase in pyknotic neurons in the CIS treatment group (p *<* 0.001). This study indicates that CIS treatment significantly impaired medulla oblongata, and combined treatment with MEL ameliorates the injury in rats.

## 1. Introduction

The medulla oblongata is located between the brain stem and the spinal cord. It is responsible for the cardiovascular-respiratory regulation system, reflexes, and functions related to the autonomic nervous system, such as circadian rhythm (Verner et al., 2008). Cisplatin (Cis-diamine-dichloro-platinum) is used in various cancer types. Clinical and experimental studies have shown that CIS treatment increases oxidative tissue damage, causes apoptosis, and increases inflammation (Cankara et al., 2021). Clinical data have indicated that one-third of cancer patients are related to CIS-induced neurotoxicity. It has been revealed that CIS crosses the blood-brain barrier and accumulates in tissues such as the hippocampus, spinal cord, and medulla oblongata at different doses and, thus, inhibits neuronal stem cell proliferation (Cavaletti, 2010) and has neurotoxic effects on the peripheral nervous system (Kandeil et al., 2020). These findings indicate that free radical formation and cell death has an essential role in CIS neurotoxicity (Kandeil et al., 2020). The cytotoxic mechanism of CIS is explained by the covalent binding of the platinum to nuclear DNA and blocking DNA replication mechanism (Cavaletti, 2010). As a result, apoptotic cell death, mitochondrial dysfunction, and neurodegeneration occur (Cavaletti, 2010; Kandeil et al., 2020). For all that, perceptual, memory, attention and dysfunction, cerebral infarctions, peripheral neuropathy may clinically occur in patients following CIS treatment, changes in quality of life (Troy et al., 2000; Kandeil et al., 2020). Although these side effects of CIS have been known recently, the molecular mechanisms underlying this outcome on the medulla oblongata are unclear.

Cytokines related to inflammation are secreted in the brain by glia and astrocytes in reply to infection, trauma, and toxic substances (Streit et al., 2004). Procaspases activated at these sites receive signals through cell surface death receptors such as fatty acid synthases (*Fas*) and tumor necrosis factor (*TNF)* receptors. Activation of proapoptotic (B-cell lymphoma-2) *Bcl-2* family members such as (Bcl-2-associated X protein**)** *Bax* leads to changes in the mitochondrial permeability and the liberation of mitochondrial cytochrome c into the cytosol (Devarajan et al., 2002).

Aquaporins (*Aqps*) are integrated membrane proteins that regulate osmolarity by mediating the bidirectional transport of water throughout cells. Toxicity of platinum in CIS content often induces increased water transport along the blood-brain barrier and astrocytes, which may have significant outcomes on the expressions of *Aqps* in the brain (Silva, 2016). *Aqp-1* and *Aqp-4* are widely expressed in the central nervous system and brain. While *Aqp-1*, which is responsible for cerebrospinal fluid production, is expressed in the epithelium of the choroid plexus, *Aqp-4* is expressed plenty on the brain-cerebrospinal fluid and blood-brain barrier (Manley et al., 2000). The specific site of *Aqp-4* suggested that *Aqp-4* has a principal function in cerebral water balance (Papadopoulos and Verkman, 2007).

Despite all these side effects, it is inevitable to treat cancerous cells with CIS; however, it is essential to develop additional treatment methods to diminish the toxic injury of CIS on other tissues. Melatonin (N-acetyl-5-methoxytryptamine) is a neuroendocrine substance produced by the pineal gland. It has been suggested as a neuroprotective agent against toxins (Beni et al., 2004). MEL’s neuroprotection ability has been demonstrated in the models of brain and spinal cord trauma (Genovese et al., 2005) and cerebral ischemia (Liu et al., 2019). MEL can undoubtedly pass the blood-brain barrier, detoxify radicals, and reach the nucleus, protecting DNA from oxidative damage. All of these are among the advantages unique to MEL (Reiter et al., 2000).

In the case of medulla oblongata injuries, no studies in the literature have evaluated the neuroprotective effect of MEL on CIS-induced toxicity. It was seen that the relationship between medulla oblongata, edema, *Aqp-1-4* channels, and MEL has not yet been fully demonstrated. Therefore, in this study, CIS-induced medullary edema and *Aqp-1-4* expression changes were examined. The contribution of apoptotic and inflammation markers to edema formation was evaluated, and the protective effects of MEL against CIS-induced toxicity was investigated.

## 2. Materials and methods

### 2.1. Drugs and chemicals

Melatonin (absolute grade, Cas number: 73-31-4, Merck) was prepared in physiological solution with 5% ethanol, and the final concentration included less than 1% ethanol. Cisplatin was obtained from Merck (Cas number: 15663-27-1) and used accordingly.

### 2.2. Experimental design

A total of 24 male Wistar albino rats were obtained from the Experimental Research Center of Çanakkale Onsekiz Mart University and were divided into four groups: i) control (C), ii) melatonin (MEL), iii) cisplatin (CIS); and iv) cisplatin + melatonin (CIS+MEL) (n = 6, randomly). All rats were kept in a light/dark (12h/12h) cycle at a standard room temperature (22±2 °C).

**Figure 1:**
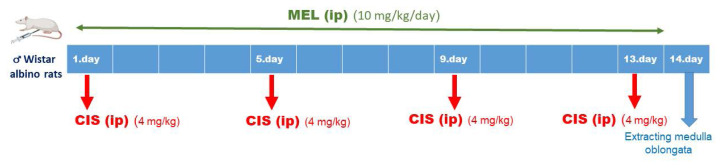
Demonstration of the CIS+MEL group experiment protocol at a timeline.

For the rats in the MEL and CIS+MEL groups, melatonin solution was prepared freshly every day as an aqueous solution containing less than 1% ethanol and administered intraperitoneally (i.p.) at a dose of 10 mg/kg/day for 13 days. For the rats in the CIS and CIS+MEL group, cisplatin solution was prepared by dissolving cisplatin in isotonic physiological serum at a treatment dose of 4 mg/kg and administered i.p. on days 1, 5, 9, and 13 (Figure 1). The physiological saline solution and the blank solution containing less than 1% ethanol were administered intraperitoneally (i.p.) to the control group rats for 13 days. On day 14, blood samples were drawn by cardiac puncture under general anesthesia. The rats were euthanized, and the medulla oblongata were harvested in all groups. This study was approved by the Experimental Animals Local Ethics Committee of Çanakkale Onsekiz Mart University (Turkey) with the decision number of 2021/09-03 related to the care and ethical use of laboratory animals. All the operations performed in this experiment were carried out in accordance with the 1964 Helsinki Declaration standards.

### 2.3. RNA isolation and qRT-PCR analysis

Total RNA was isolated from the medulla oblongata obtained from each rat following the kit protocol (PureLink Thermo Fisher Scientific). The quality and amount of RNA were measured at 260 nm and 280 nm (ND-1000 spectrophotometer). The synthesized cDNA samples were used for quantitative real-time PCR (qRT-PCR) using the StepOne Real-Time PCR System (Thermo Fisher Scientific). The TaqMan probe details of the primers used in the qRT-PCR experiments are given in Table.

### 2.4. Histopathological analysis

Medulla oblongata was fixed in a formaldehyde solution at room temperature for 72 h. After fixation, tissue was washed with tap water overnight and dehydrated in ethanol, cleared in xylene, and embedded in paraffin wax. Paraffin blocks were cut at a 4-μm thickness using a microtome and stained with hematoxylin and eosin. Slides were then examined using a light microscope and the ZEISS ZEN image analysis software system (Zen 2.6, Zeiss AG). Histopathological degenerations were scored as none (0), mild (1), moderate (2), and severe (3) (Kazak et al., 2021).

### 2.5. Statistical analysis

All the data were analyzed with SPSS v20.0 for Windows (Armonk, New York, USA: IBM Corp.) and evaluated with the Kolmogorov–Smirnov test and the Shapiro–Wilk test to define the normal distribution. A parametric test was performed for the gene expression, and groups were compared by a one-way ANOVA test, followed by the post hoc Tukey’s test. Histopathological data were analyzed by nonparametric test, and comparison between groups was made using Mann–Whitney U tests. A *p*-value of <0.05 was considered statistically significant. The relative quantification of the gene expression was evaluated by the comparative cycle threshold (CT) (2^−ΔΔCt^) method [ΔΔCt = (Ct _Target gene_−Ct _reference gene_)].

## 3. Results

The medulla oblongata of Wistar albino rats was examined in terms of genes encoding inflammation (*TNF-α*, *IL-6*), apoptosis (*Bax*, *Bcl-2, Casp-3*), and Aquaporin water channels (*Aqp-1*,*- 4)* following administration of toxic doses of CIS. Additionally, the neuroprotective role of MEL against CIS-induced medulla oblongata injury was investigated histopathologically.

### 3.1. Analysis of mRNA expression

**Figure 2:**
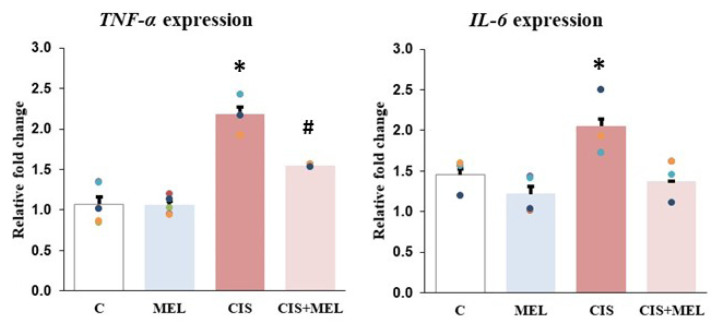
Between-group differences in the gene expression levels of *TNF-a* and *IL-6.* The results were represented after the mRNA levels were normalized with *β-actin.* All the results were shown as mean ± standard error for six rats in each group. Relative fold change of the *TNF-a* and IL-6*: compared to C, MEL, and CIS+MEL groups (p < 0.0001), #: compared to C and MEL groups (p < 0.001).

At the end of the 13-day experiment period, the expression changes of *TNF-α*, *IL-6*, *Bax*, *Bcl-2*, *Casp-3*, *Aqp-1*, and *Aqp-4* genes in medulla oblongata were examined. *TNF-α, IL-6* expression changes were increased in the CIS group. *TNF-α* and *IL-6* expression changes in C and MEL groups were found to be parallel to each other (p < 0.001) (Figure 2).

**Figure 3:**
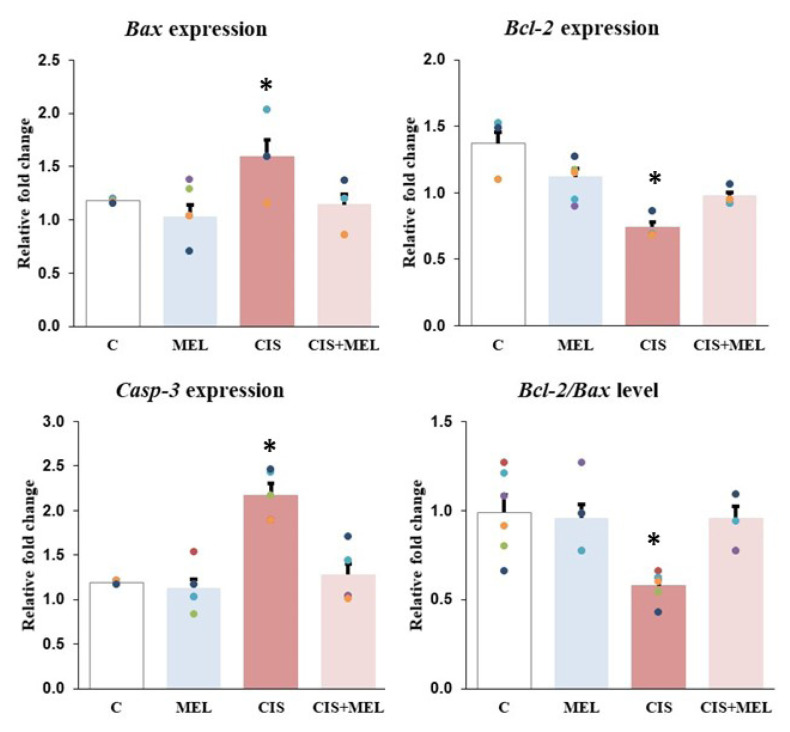
Between-group differences in the gene expression levels of apoptosis-related genes *Bax*, *Bcl-2*, *Casp-3*, and *Bcl-2/Bax* levels. The results were represented after the mRNA levels were normalized with β-actin. All the results were represented as mean ± standard error for six rats in each group. Relative fold change of the Bax*: compared to MEL (p = 0.008) and CIS+MEL groups, (p = 0.004). Bcl-2*: compared to C (p < 0.001), MEL (p < 0.001) and CIS+MEL (p = 0.004). Casp-3*: compared to C (p = 0.003), MEL and CIS+MEL (p = 0.006). *Bcl-2/Bax* level *: compared to C (p = 0.003), MEL and CIS+MEL (p = 0.006).

The apoptotic gene expression analysis results revealed major changes in *Bax*, *Bcl-2*, and *Casp-3* expression. The effect of CIS on the expression of *Bax*, *Bcl-2*, and *Casp-3* genes was significantly different between the group that did not receive CIS, and the group that received only MEL. *Bax* and *Casp-3* expression changes were up-regulated in samples belonging to the CIS group with CIS administration, while *Bcl-2* expression changes were down-regulated. *Bax*, *Casp-3* and *Bcl-2* values were statistically significant when compared with C, MEL, and CIS+MEL groups (p = 0.004, p = 0.006, p = 0.004, respectively). It was found that *Bax* and *Casp-3* gene expression levels were statistically decreased in the CIS+MEL group following MEL administration (p = 0.004) (Figure 3). While the ratio of *Bcl-2/Bax*, which is important in the apoptotic process, was quite close to each other in the C and MEL groups, it was found to be significantly lower in the CIS group compared to the other groups (p < 0.05) (Figure 3).

**Figure 4:**
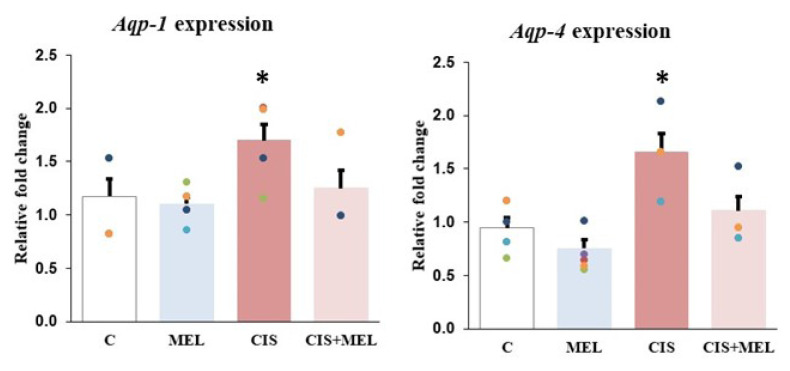
Between-group differences in the gene expression levels of aquaporin channel genes, *Aqp-1* and *Aqp-4*. The results were represented after the mRNA levels were normalized with **β-actin**. All the results were represented as mean ± standard error for six rats in each group. Relative fold change of the Aqp-1*: compared to CIS+MEL group (p = 0.003) and Aqp-4*: compared to C (p = 0.003), MEL (p < 0.001) and CIS+MEL (p = 0.025).

*Aqp-1* gene expression was found to be decreased in the C, MEL, and CIS+MEL groups compared to the CIS group, and there was a statistical significance only between the CIS and CIS+MEL groups (p = 0.003). While *Aqp-4* gene expression, which has a wide distribution in the brain, did not change significantly in the C and MEL groups (p = 0.686), it increased 2-fold in the CIS group compared to the C and MEL groups (p = 0.003 and p < 0.001, respectively). On the other hand, the expression level of the *Aqp-4* gene was increased in the CIS group compared to the CIS+MEL group (p = 0.025) (Figure 4).

### 3.2. Histopathologic evaluation

**Figure 5:**
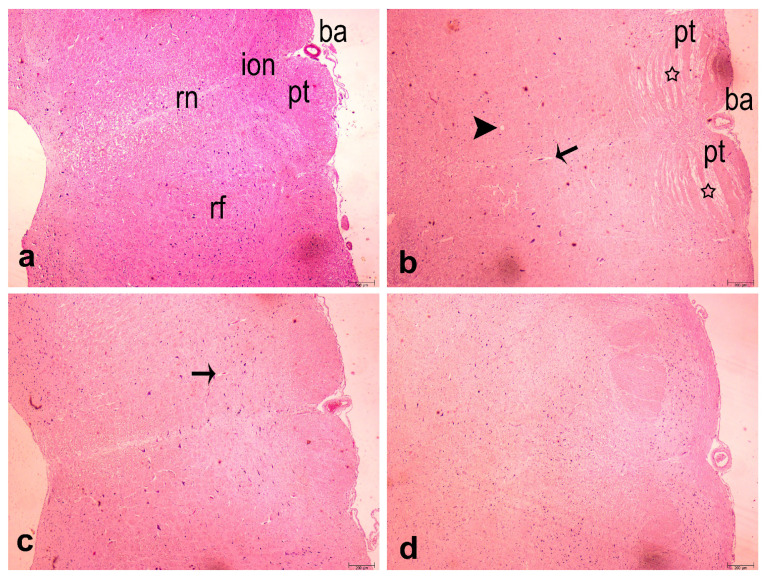
Histological examination of the medulla oblongata stained with hematoxylin-eosin at low magnification (40×). a: control (C), b: CIS, c: CIS+MEL and d: MEL groups. In the CIS group, edema and degeneration were observed in the pyramidal tracts (star). Cerebral edema (arrowhead) and extravascular edema (thin arrow) histopathological changes were also observed in the CIS group (star: pyramidal tract degeneration, arrowhead: cerebral edema, arrow: extravascular edema, ba: basilar artery, pt: pyramidal tract, ion: inferior olivary nucleus, rn: reticular nuclei, rf: reticular formation, scale bar: 200 μm, magnification: 40×, paint: hematoxylin-eosin).

A centrally located nucleus and a clean structure were observed in the neurons in the C and MEL groups (Figure 5). No significant difference was observed within the morphological boundaries, and the typical tissue architecture was preserved (Figure 5a). In the CIS group, hemorrhage, edema, and perivascular edema showed a more intense distribution than the other groups (Figure 5b). In addition, cells with chromatolysis nuclei, degenerated neurons, and pyknotic nuclei were present. Several levels of intracellular and perivascular edema and degenerative changes were observed in all cases belonging to the CIS+MEL group (Figure 5c). Again, this group had fewer chromatolysis and degenerate neurons than the CIS group and rarely dispersed pyknotic nuclei. C and MEL groups showed typical tissue structure (Figure 5a, 5d). Hemorrhage, perivascular edema, and edema histopathological degenerations were seen in the CIS group (p < 0.0001). Histopathological changes were milder in the CIS+MEL group (Figure 5).

The histologic evaluation shows segments of pyramidal tracts, inferior olivary nuclei, raphe nuclei, and reticular formation in medulla oblongata specimens belonging to the groups. The inferior olivary nucleus is detected ventromedially in the caudal section of the medulla oblongata, lateral to the pyramidal tracts. Medially, the reticular formation extends through the brainstem center, midbrain, and medulla oblongata, and the serotonin-synthesizing raphe nuclei subregion of the reticular formation. Edema and degeneration findings were observed, especially in the pyramidal tract in the CIS-administered group (star, Figure 5b). Cerebral (arrowhead) and extravascular edema (thin arrow) findings were observed in the CIS group (Figure 5b). In the group treated with CIS+MEL, degenerations in the pyramidal tract decreased, and, rarely, extravascular edema (thin arrow) was observed (Figure 5c). The histological structure was similar to the control group in the MEL-administered group, and no histopathological changes were found (Figure 5a and 5d).

**Figure 6:**
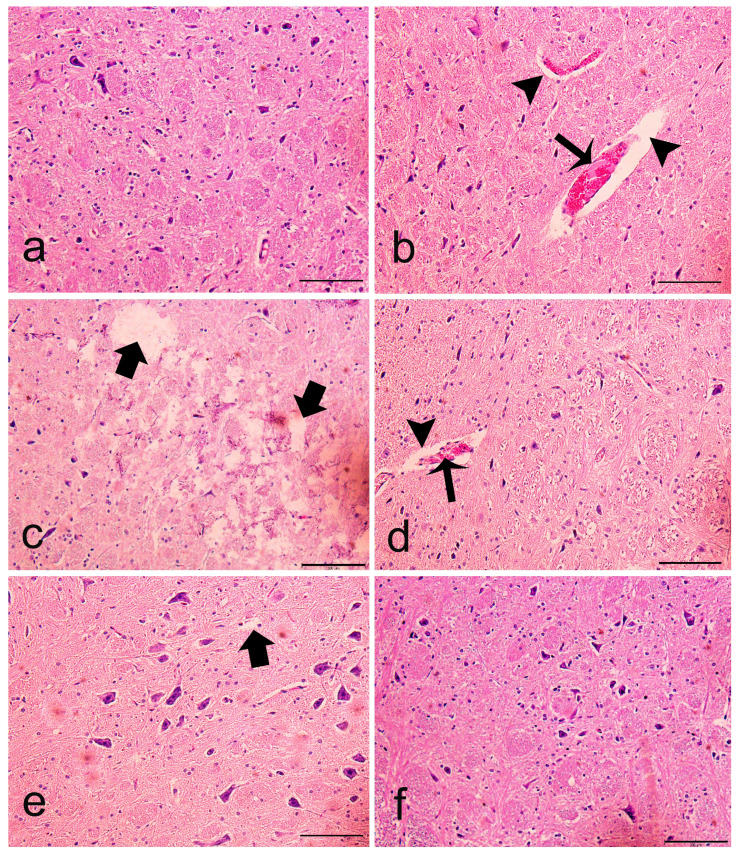

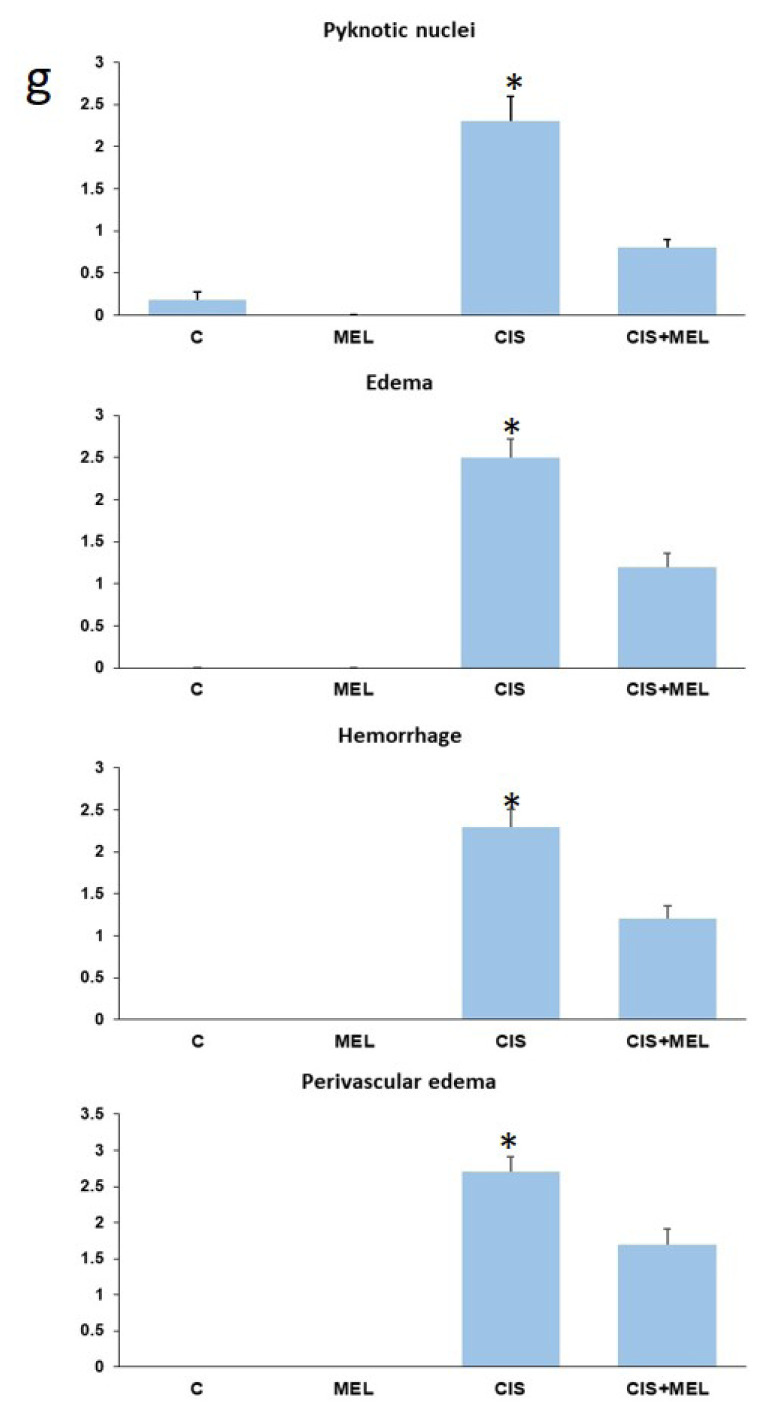
Histopathological examination of MEL on CIS-induced neurotoxicity. a: control (C), b, c, and d: CIS, e: CIS+MEL and f: MEL groups. (arrowhead: perivascular edema, thin arrow: hemorrhage, thick arrow: edema, tailed arrow: pyknotic nuclei, dye: hematoxylin and eosin, magnification: 200×, scale bar: 200μm). g: In terms of pyknotic nuclei, edema, hemorrhage, perivascular edema changes, the scores were none (0), mild (1), moderate (2), and severe (3). Bars represented as mean ± standard error (n = 6). *: Mann–Whitney U-test. p < 0.001.

Figure 6 shows the detailed histopathological examination of the medulla oblongata. Numerous blood vessels, neuroglia cells and motor neurons, and myelin sheath structures were observed in microscopic examination in the control group (Figure 6a). Histopathological changes were observed in the CIS administered groups. Evidence of extravascular edema was observed in almost all blood vessels (Figure 6b, arrowhead). Hemorrhage was evident in the blood vessels (Figure 6b, thin arrow). In addition, signs of severe edema were observed in the medulla oblongata (Figure 6c, thick arrow). Pyknotic nuclei were noted in the edematous areas (Figure 6d, tailed arrow). In the group treated with MEL in addition to CIS, the histopathological findings were alleviated, and the findings of cerebral edema were weak in some areas (p < 0.001) (Figure 6e). The MEL group showed a typical tissue structure (Figure 6f). It was evaluated that edema in the medulla oblongata increased markedly in the rats which were given cisplatin, however pyknotic nuclei, hemorrhage, and perivascular edema scores statistically significantly decreased when melatonin was given (p < 0.001) (Figure 6g).

## 4. Discussion

In this study, we aimed to investigate a) the histopathological changes including the formation of edema, pyknotic nuclei, and hemorrhage in the medulla oblongata after CIS administration, b) the alterations in the expressions of *Aqp-1* and *Aqp-4* genes, c) the effects of MEL as a protective co-treatment by reducing edema and regulating the gene expressions.

**Figure 7:**
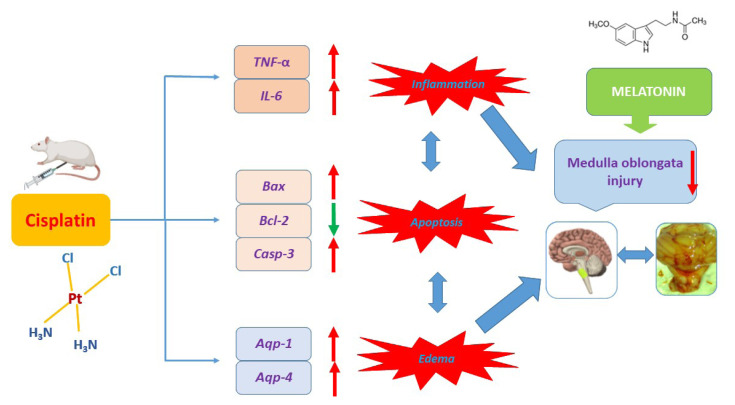
Schematic summary of CIS-induced neurotoxicity in medulla oblongata.

Changes in the expressions of selected genes and histopathological examinations revealed that *Aqp-1* and *Aqp-4* expressions and formation of edema increased in the CIS-administered group and significantly decreased after MEL administration. In addition, MEL was found to regulate neurogenic inflammation (*TNF-α* and *IL-6*) and the expressions of apoptosis markers (*Bax*, *Casp-3*, and *Bcl-2*) induced by CIS. Based on perusal of literature, this is the first study examining *Aqp-1* and *Aqp-4* changes in CIS-induced neurotoxicity in the medulla oblongata and reveal the effects of MEL on these changes (Figure 7).

Neurodegenerative effects of CIS may develop due to the increased oxidative stress and inflammation (Cavaletti, 2010; Cankara et al., 2021). This stress may impair energy balance by inducing neuroinflammatory pathways (Naeem et al., 2021). It also mediates the release of cytokines such as *TNF-α* and *IL-6* (Ali et al., 2020). Zakria et al (2021) found that *TNF-α* and *IL-1β* levels in the hippocampus and frontal cortex of mice were increased in the CIS-administered group, and this inflammation decreased with MEL treatment. Similar to the results of Zakira et al. (2021), in this study also *TNF-α* and *IL-6* expression levels in the medulla oblongata increased significantly in the CIS-administered group, and MEL treatment was found to reduce inflammation. These results showed that MEL reversed oxidative stress in the medulla oblongata and regulated MEL inflammatory mediators (*TNF-α* and *IL-6*). It has been suggested that MEL has antiinflammatory actions by interacting directly with specific binding sites found in lymphocytes and macrophages (Esposito and Cuzzocrea, 2010)

One of the adverse effects of CIS is inhibition in DNA replication process (Cavaletti, 2010). CIS-induced toxic stress prompts multiple signal transduction pathways that may join apoptosis or chemotherapy resistance (Manohar et al., 2014). Members of the Pro (*Bax*) and antiapoptotic (*Bcl*) protein family have been found to arrange the mitochondrial function for apoptosis vigorously, and overexpression of *Bcl-2* inhibits apoptosis (Yip and Reed, 2008). In this study, due to the CIS (4 mg/kg) administration in 4-day intervals, *Bcl-2* expression was down-regulated in the CIS applied group. In contrast, *Bax* and *Casp-3* genes’ expressions were upregulated and significantly increased compared to the control group. Manohar et al. (2014) tested 12 mg/kg acute CIS administration and found that hippocampal cell proliferation was inhibited two days after administration, and the expressions of apoptotic genes are altered. These results showed that MEL reduced oxidative stress by down-regulating the expressions of apoptotic *Bax* and *Casp-3* genes and similarly positively modulated *Bcl-2* gene expression. It’s reported that MEL has ability to reduce the endoplasmic reticulum and oxidative stress mechanisms by regulating these apoptotic and autophagic processes (Fernandez et al., 2005). Our findings about the development of neurotoxicity during CIS treatment include both intracellular and perivascular degeneration. Al-Gholam and Issa (2020) reported that MEL reduces the neurotoxicity caused by cypermethrin, and its concomitant usage preserves histomorphology. Similarly, Rao and Purohit (2011) showed that MEL administration (5 mg/kg/day) ameliorated the pathological changes observed in many tissues and medulla oblongata in rat central nervous system (CNS) exposed to mercury toxicity. The reason for this was found to be the potential neuroprotective effect of MEL against apoptosis in the brainstem of rats.

When CIS enters the cerebral tissues, it is more difficult to remove the platinum accumulated in the tissues than other tissues because the blood-brain barrier is disrupted (Namikawa et al., 2000). Therefore, the development of edema in the tissue increases as the blood-brain barrier deteriorates. This edema is explained by increased water transport across the blood-brain barrier to brain tissue, swelling of cells, and increased head pressure. In the treatment of neurotoxicity, antioxidant compounds must cross the blood-brain barrier for the drug to be administered peripherally. As MEL can cross the blood-brain barrier, maintain healthy neuronal organization, and not have any toxic side effects, it could be a potential cotreatment agent for CIS administration. In this study, histopathological degenerations characterized as medulla oblongata degeneration, perivascular, and cerebral edema were observed in the CIS-treated rat group. MEL administration especially caused a significant decrease in edema and perivascular level. It has been reported that MEL provides this effect by activating free radical production by microglial cells, regulating membrane fluidity, and reducing edema (Esposito and Cuzzocrea, 2010).

*Aqp*s are integral membrane proteins and possess important roles in transcellular and transepithelial water transport (Verkman, 2005). Several *Aqp*s have been identified that are thought to be involved in producing and absorbing cerebral fluid in the CNS. *Aqp-1* is located in the apical membrane of the choroid plexus epithelium and the ependyma and pia in the CNS, while *Aqp-4* is the primary water channel expressed in glial cells (Silva, 2016). *Aqp-4* is involved in cerebrospinal fluid secretion (Verkman, 2005), cerebral water balance, brain edema development, and regulation of glial cell migration (Oshio et al., 2005). Recent studies have reported that *Aqp-4* can act as an osmosensor in normal and pathological conditions (Agre et al., 2002). Besides, there is evidence that *Aqp-4* is related to water transport in and out of the brain and spinal cord, neuroexcitation, and astrocyte movement after degeneration (Verkman and Anderson, 2014). Further, studies show that *Aqp-4* facilitates the outflow of water from the brain in vasogenic edema (Papadopoulos et al., 2004). Toxic amounts of metal ions in the CNS cause disturbances in brain metabolism and increases in water permeability. These changes are mainly related to increased *Aqp-4* expression in astrocytes surrounding the blood-brain barrier, oxidative stress in neurons and astrocytes, and brain swelling leading to neurodegeneration (Akdemir et al., 2014; Silva et al., 2016). In accordance with this outcome, it was determined that edema of brain tumors increased in chemotherapy treatments, and edema formation occurred in the brain after high-dose local carmustine treatment (Platten, 2012). In a study, *Aqp-4* knockout mice have been shown to survive much better than wild-type mice in a model of brain edema caused by acute water intoxication. Cerebral edema was reduced by 35% in these mice (Manley, 2000). Moreover, *Aqp-4*-deficient mice had reduced brainstem response potential and significantly increased seizure threshold. These findings indicate that *Aqp-4* is involved in the regulation of neural signaling (Manley, 2000). Additionally, Akdemir et al (2014) found that *Aqp-4* inhibition could improve the neurological outcome and increase the chance of survival following global ischemia. In the present study, CIS treatment caused a significant increase in *Aqp-4* gene expressions compared to the CIS-MEL group, and the treatment of MEL redounded in a significant decrease in *Aqp-4* gene expression compared to the CIS group. We also found a correlation between edema formation and regulation of Aqp-4 expression. Costa et al. (2007) stated that *Aqp-1* and *Aqp-4* were overexpressed in a model of transmissible spongiform encephalopathies, and overexpression of *Aqp* in glial cells led to deterioration in water and ion balance and then triggered neural dysfunctions and vacuole formation. In this study, *Aqp-1* expression levels, which were increased with CIS, were also decreased by MEL treatment. At the same time, it was observed that edema and cell damage were reduced in tissue architecture. *Aqp-4* expression is notably changed in investigational models of brain injury and edema, and transgenic mice lacking *Aqp-4* are moderately preserved from brain swelling in reaction to acute hyponatremia and ischemic stroke. Therefore, *Aqp*s and promoter of brain *Aqp* expressions are potential treatment targets in the invention of compounds being researched for the therapy of brain edema. A study determined that MEL has a protective effect against edema formation in spinal cord damage by affecting the regulation of *Aqp-4* expression (Liu et al., 2005).

The neuroprotective properties of MEL are well known (Reiter et al., 2000), but, until now, no study has investigated the *Aqp-1* and *Aqp-4* gene expression changes in the medulla oblongata section and the effect of MEL on that specific location. Besides, no study has evaluated the relationship between medulla oblongata degeneration, edema, apoptosis, and inflammation. However, in this study, we found that MEL regulated the *Aqp-1* and *Aqp-4* gene expressions, reduced inflammation and edema, and, hence, neurodegenerative formations were inhibited in the medulla oblongata.

As a limitation, only light microscope results are available. Then, the antioxidant parameters need to be analyzed. Changes in protein and gene expressions can also be verified by western blot and immunohistochemical analysis.

## 5. Conclusion

It has been determined that antioxidant adjunct is essential in increasing the response of cancer patients to chemotherapy and their quality of life, and MEL is a potential agent with its neuroprotective properties. Thanks to this study, the following findings have been identified and listed as a summary:

1. In the experimental rat model designed by applying treatment dose of CIS, structural anomalies occurred in the medulla oblongata according to histopathological and gene expression results.
2. MEL was found to regulate and improve the expression of both apoptotic genes and inflammatory genes and genes encoding the water channels (*Aqp-1* and *Aqp-4*) and edema.
3. It has been determined that more studies should be performed to examine the relationship between the medulla oblongata, edema, and other *Aqp* water channels.
4. We suggested that the effects of MEL on edema in the medulla oblongata and on *Aqp* channels should be tested further by utilizing *Aqp* channel inhibitors.

## Figures and Tables

**Table:** Genes, primers, and ID numbers used in the qRT-PCR.

Primer	Gene	ID Number

Inflammation marker	*TNF-α*	Rn01525859_g1

	*IL-6*	Rn01410330_m1

Apoptosis marker	*Bax*	Rn01480161_g1

	*Bcl-2*	Rn99999125_g1

	*Casp-3*	Rn00563902_m1

Water channels	*Aqp-1*	Rn00562834_m1
	*Aqp-4*	Rn01401327_s1

Endogen control	*β-actin*	Rn00667869_m1

PCR conditions: 1 cycle of 2 min at 50 °C and 2 min at 95 °C for polymerase activation, followed by 40 cycles of denaturation at 95 °C for 15 s, annealing and extension at 60 °C for 30 s.
